# Climate emergency and decent work

**DOI:** 10.5271/sjweh.4162

**Published:** 2024-05-01

**Authors:** Fernando G. Benavides, George L. Delclos

**Affiliations:** 1 Occupational Health Research Center (CiSAL), MELIS-Universitat Pompeu Fabra, Barcelona, Spain.; 2 IMIM-Hospital del Mar, Barcelona, Spain.; 3 CIBER de Epidemiología y Salud Pública, Instituto de Salud Carlos III, Madrid, Spain.; 4 Southwest Center for Occupational and Environmental Health, The University of Texas Health Science Center School of Public Health, Houston, USA.

The climate crisis and loss of biodiversity, two closely related threats to human and planetary health, meet the criteria for the World Health Organization (WHO) to declare an international public health emergency, as occurred with COVID-19 (1), and urged by numerous scientific journals (2).

Attaining decent work, understood as “opportunities for women and men to work in conditions of freedom, equity, security and human dignity” (3), in the context of the climate emergency, creates a paradox for worker health. Outdoor workers (notably those in agriculture and construction), many of them informal workers, are among the populations most vulnerable to climate-related hazards. Simultaneously, they are inevitably at risk of exposure due to their role in maintaining the economy and functioning of society. A similar situation happened during the pandemic with essential workers (4). The WHO declaration of a public health emergency helped manage that global crisis.

## A consequence of the industrial revolution

The current climate crisis is a direct consequence of the Industrial Revolution where key processes emerged to explain the current situation: the appearance of wage labor and the working class, with consumerism as a basic economic driver, and the exploitation of natural resources – especially fossil fuels – in their own territories and in the colonies.

The extension of this capitalist model of society to virtually the entire planet is a reality. Now, we see how this economic system has brought both great harm and significant benefits.

Since its beginning, capitalism has wrought great suffering for people, masterfully described, among others, by Fredrich Engels in the Manchester of 1845 (5) or the London of 1838 in Charles Dickens' *Oliver Twist* (6). Although working conditions have since improved in many countries, there are still unbearable examples worldwide of worker exploitation and suffering. Among them, child labor, where 70% are working in agriculture (7) or some underregulated platform work (8), in a context of ever-increasing social inequalities (9).

On the other hand, due to improved working and life conditions, there has also been an extraordinary increase in the world population, from one billion at the beginning of the 19^th^ century to approximately eight billion today, leading to a linear increase in life expectancy at birth, which doubled globally between the beginning of the 20^th^ century and the present. In 2015, the Lancet Commission on Planetary Health (10) pointed out that never before has humanity faced such an unintended paradox. While human well-being has been improving, the planet has been degrading. A contradiction that can no longer be sustained.

We have lived as if our planet's resources are unlimited. Based on comparisons to average temperature readings of the planet between 1850 and 1900, the United Nations International Panel on Climate Change estimated in its latest report that temperatures increased by 1.1° C between 2011 and 2020. This increase is very close to the 1.5° C established by the 2015 Paris Agreement as the limit beyond which climate impacts may become irreversible. Beyond any reasonable doubt, this is mostly attributable to the greenhouse effect produced by CO_2_ emissions, a consequence mainly of human activity and our way of living initiated by the Industrial Revolution.

This global increase in temperature, with heat waves, floods and other extreme temperature events as its most obvious manifestations, is already having effects on worker health (12, 13). Climate change is also having effects on the economy and the labor market, both in the primary (agriculture and fishing) and services (tourism) sectors, with reductions in productivity and employment. Estimates from the European Commission reveal an average loss of 3% of GDP among EU countries between 1980 and 2020 (14).

Simultaneously, we should not forget that the capitalist society that emerged from the Industrial Revolution is based, among other pillars, on full or near full employment. As such, wages represent the main economic resource for the majority of people, in addition to being the primary source of wealth generation for society, on whose income and taxes the welfare state was built. Of course, employment means much more than wage earning, as it plays a fundamental role in the social processes that sustain human dignity and social cohesion (15). However, only approximately 50% of the employed population, mainly in high-income countries, enjoy decent employment with a living wage and social rights (16).

The resulting Gordian knot before us is enormous, with humanity facing the climate emergency and trying to move from fossil fuels to renewable energy sources, while simultaneously seeking to maintain and increase decent employment for all Earth's inhabitants, boosting the welfare states at the same time (17).

## Controlling climate-related hazards and just green transition

The alternatives proposed to escape this crossroads vary between those that propose a new paradigm, which radically changes the current economic model, betting on measures that break drastically from the capitalist economy (18), versus a gradual process, supported by mitigation, adaptation, and compensation policies (19).

Favoring this second alternative, but without ruling out the need to profoundly change human consumption patterns with important repercussions on the productive system (energy, transportation, food, etc.), gradualist policies will also directly or indirectly impact employment and working conditions during the transition from carbon emission energy to green energy.

To cope with this urgent situation, specific control measures have been proposed over the last few decades. Schulte and colleagues have systematically reviewed the literature (20–22), identifying new and exacerbated old climate-related hazards such as extreme temperatures, air pollution, ultraviolet radiation, natural disasters, biological hazards, indoor air quality, etc., and they also assessed the impact of employment transition and economic burden on occupational health equity and mental health. On this basis, the US National Institute for Occupational Safety and Health has elaborated recommendations to mitigate and control the impact of several climate-related hazards on worker health and well-being (23). Similarly, the EU Agency for Safety and Health at Work has published guidelines for heat at work (24). Going further, some governments, such as Spain, have begun regulating and enforcing specific measures (25).

Implementation of these workplace preventive measures to mitigate the impact of climate change is the responsibility of employers, with full participation of workers. Devoting resources to hazard recognition; performing risk assessments to identify which workers are most vulnerable to climate change-related hazards; and implementing a control strategy with policies, procedures, equipment, and work organization changes aiming to eliminate or minimize the impact of these hazards can improve employer preparedness (26).

Adaptation policies to reduce emissions of CO_2_ and other gases that are driving the greenhouse effect, still with limited results, could mean a loss of six million jobs worldwide, according to estimates of the International Labor Organization (ILO) (27). This same estimation predicts a promising creation of 24 million jobs, mainly in economies emphasizing recycling and reutilization of manufactured products (the so-called “circular economy”), infrastructure construction, development of renewables and energy efficiency. Also, during this transition, new forms of work will emerge (e.g., human-robot interfaces and artificial intelligence), and with them the need to train workers, both new and existing, to adapt to those new forms of work.

While waiting for positive results from mitigation and adaptation policies, a just transition to a green economy must simultaneously incorporate compensation policies. To achieve this, it is essential to strengthen social protection systems, a cornerstone of decent employment. For example, there were measures adopted during the pandemic, such as temporary employment regulation for employees or benefits covering the cessation of activity of the self-employed. Similar compensation measures may help workers affected by mitigation and adaptation policies during a transition phase, possibly to a lesser degree than in the pandemic, but lasting longer.

In summary, as was the case in the most recent public health emergency, the COVID-19 pandemic, declaring the climate emergency as an international public health emergency by the WHO could play a critical role in managing this new global health crisis. Research programs, supported by global occupational health surveillance systems, to monitor the effectiveness of mitigation, adaptation and compensation measures are urgent.
